# Assessment of Potential Risk Factors for the Development of Persistent Postural-Perceptual Dizziness: A Case-Control Pilot Study

**DOI:** 10.3389/fneur.2020.601883

**Published:** 2021-01-21

**Authors:** Aaron Trinidade, Paula Harman, Jon Stone, Jeffrey P. Staab, Joel A. Goebel

**Affiliations:** ^1^Southend University Hospital NHS Foundation Trust, Southend-on-Sea, United Kingdom; ^2^Centre for Clinical Brain Sciences, University of Edinburgh, Edinburgh, United Kingdom; ^3^Mayo Clinic, Rochester, MN, United States; ^4^Washington University in St. Louis School of Medicine, St. Louis, MO, United States

**Keywords:** state anxiety, neuroticism, body vigilance, illness perceptions, PPPD

## Abstract

**Objectives:** (1) To assess whether neuroticism, state anxiety, and body vigilance are higher in patients with persistent postural-perceptual dizziness (PPPD) compared to a recovered vestibular patient group and a non-dizzy patient group; (2) To gather pilot data on illness perceptions of patients with PPPD.

**Materials and Methods:** 15 cases with PPPD and two control groups: (1) recovered vestibular patients (*n* = 12) and (2) non-dizzy patients (no previous vestibular insult, *n* = 12). Main outcome measures: Scores from the Big Five Inventory (BFI) of personality traits, Generalized Anxiety Disorder - 7 (GAD-7) scale, Body Vigilance Scale (BVS), Dizziness Handicap Inventory (DHI), modified Vertigo Symptom Scale (VSS) and Brief Illness Perception Questionnaire (BIPQ).

**Results:** Compared to non-dizzy patients, PPPD cases had higher neuroticism (*p* = 0.02), higher introversion (*p* = 0.008), lower conscientiousness (*p* = 0.03) and higher anxiety (*p* = 0.02). There were no differences between PPPD cases and recovered vestibular patients in BFI and GAD-7. PPPD cases had higher body vigilance to dizziness than both control groups and their illness perceptions indicated higher levels of threat than recovered vestibular patients.

**Conclusion:** PPPD patients showed statistically significant differences to non-dizzy patients, but not recovered vestibular controls in areas such as neuroticism and anxiety. Body vigilance was increased in PPPD patients when compared with both recovered vestibular and non-dizzy patient groups. PPPD patients also exhibited elements of negative illness perception suggesting that this may be the key element driving the development of PPPD. Large scale studies focusing on this area in the early stages following vestibular insult are needed.

## Introduction

The diagnosis *persistent postural-perceptual dizziness (PPPD)* entered the 11th edition of the World Health Organization's International Classification of Diseases (ICD-11 beta draft) in 2015 following a consensus document on its diagnostic criteria created by Bárány Society for the International Classification of Vestibular Disorders (ICVD) and the criteria for its dignosis are outlined in Table 1 (1–3).

**Table 1:** Criteria for the diagnosis of persistent postural-perceptual dizziness (PPPD) as outlined by the Committee for the Classification of Vestibular Disorders of the Bárány Society (CCBS) (1).

Criteria^*^	**Description**	**Qualifiers**
A	One or more symptoms of dizziness, unsteadiness, or non-spinning vertigo are present on most days for 3 months or more	1. Symptoms last for prolonged (hours long) periods of time but may wax and wane in severity 2. Symptoms need not be present continuously throughout the entire day
B	Persistent symptoms occur without specific provocation, but are exacerbated by three factors:	1. Upright posture, 2. Active or passive motion without regard to direction or position, or 3. Exposure to moving visual stimuli or complex visual patterns
C	The disorder is precipitated by conditions that cause vertigo, unsteadiness, dizziness, or problems with balance including acute, episodic, or chronic vestibular syndromes, other neurological or medical illnesses, or psychological distress	1. When the precipitant is an acute or episodic condition, symptoms settle into the pattern of criterion A as the precipitant resolves, but they may occur intermittently at first, and then consolidate into a persistent course 2. When the precipitant is a chronic syndrome, symptoms may develop slowly at first and worsen gradually
D	Symptoms cause significant distress or functional impairment	
E	Symptoms are not better accounted for by another disease or disorder	

* *All five criteria A–E must be fulfilled to make the diagnosis of PPPD*.

**Figure 1:**
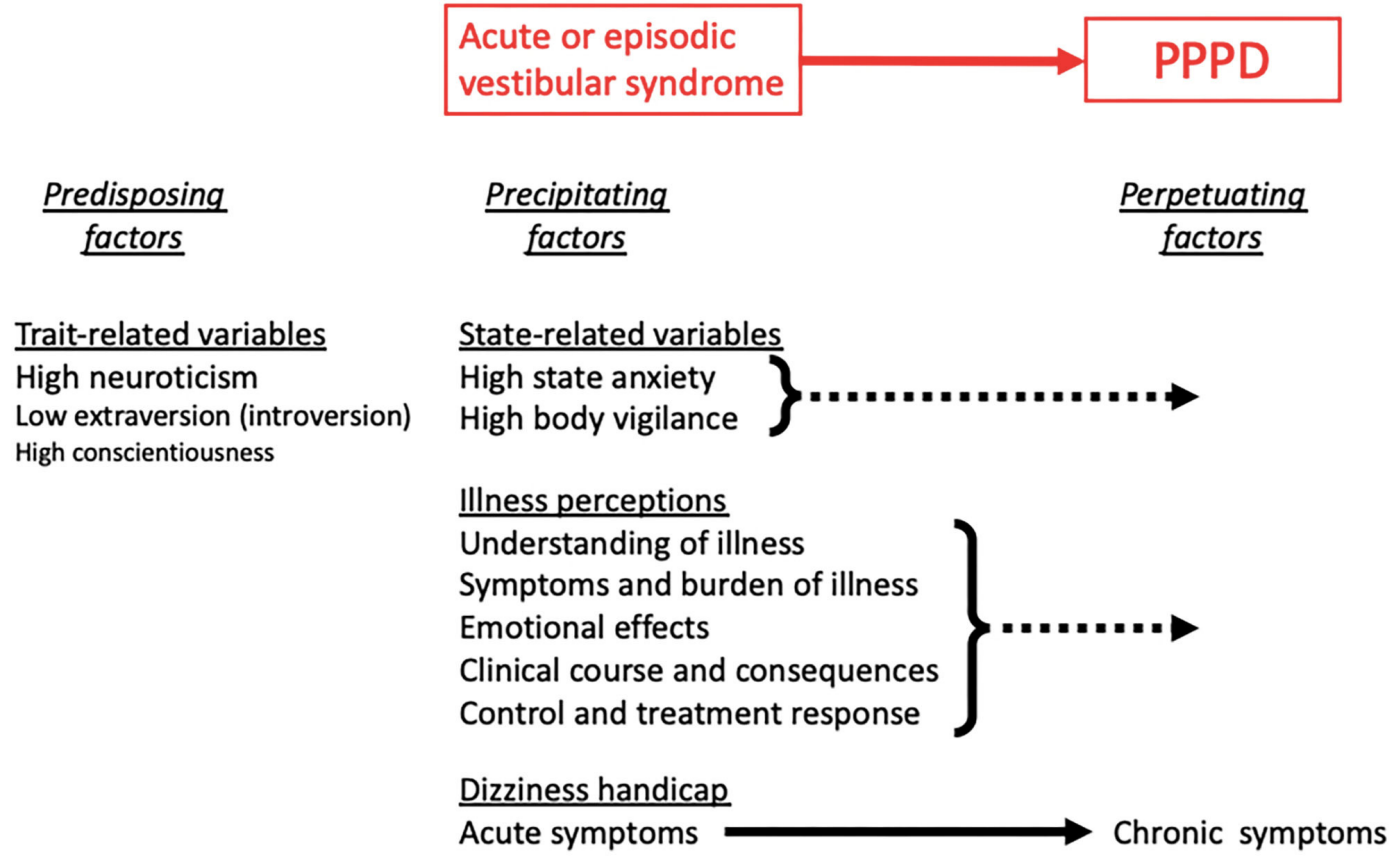
Potential risk factors for PPPD include, from left to right, anxiety-related personality traits (primarily neuroticism and introversion) that predate the onset of vestibular symptoms, high levels of state anxiety and body vigilance that coincide with the onset of vestibular symptoms, and adverse illness perceptions and dizziness-related handicap that emerge as the course of illness progresses toward PPPD rather than recovery.

PPPD is a relatively new diagnosis and to date it is still not clear what predisposes some people to it following known triggers such as acute, episodic, or chronic vestibular syndromes, other neurological or medical illnesses, or psychological distress. Several authors have shown that acute anxiety and body vigilance predicted chronic dizziness after acute vestibulopathies (4–8). A prospective study found that psychological distress predicted severity of dizziness-related handicap among patients with various vestibular disorders in the 12 months following tertiary consultation (9). Neuroticism, the personality trait tendency to experience negative emotions or psychological distress in response to life events, has also been identified as a possible predisposing risk factor in both PPPD and chronic subjective dizziness (CSD), one of PPPD's precursors (10–12). In addition patients' appraisals and perceptions of their illness have been shown to influence outcomes in a range of other medical conditions including vestibular disorders (13, 14). (Figure 1). Apart from one study of neuroticism, however (10), these investigations were conducted prior to publication of the ICD-11 and ICVD definitions of PPPD or included patients with combinations of structural, metabolic, psychiatric, and functional causes of vestibular symptoms. As such, they offer data to formulate hypotheses about roles that psychological variables and illness perceptions may play in the developement of PPPD. Confirmation or refutation of those hypotheses requires investigations that include patients explicitly diagnosed with PPPD and carefully selected comparison groups. In addition, it is yet to be deterimined if any of these factors are associated strongly and uniquely enough with the onset of PPPD to be useful for early detection of the disorder.

As a first step in validating hypotheses about the relationship of psychological variables specifically to PPPD and with the intent of designing a prospective trial to predict patients at risk for developing this disorder, the primary aim of this current study was to gather pilot data to test the hypothesis that the frequency of anxiety-related variables is higher in patients who meet ICVD criteria for PPPD compared to those who suffered acute vestibulopathies but did not develop PPPD and patients without a history of dizziness who were receiving treatment for other medical conditions. The second aim was to gather pilot data on illness perceptions in patients with PPPD. Findings of an increased prevalence or severity of anxiety-related variables or adverse illness perceptions would provide an impetus for conducting fully-powered, prospective studies with the aim of identifying a risk profile for PPPD that could guide early interventions for patients susceptible to developing this burdensome chronic dizziness condition.

## Methodology

The study was conducted as a case-control observational study (www.clinicaltrials.gov, reference NCTO3930485, with ethical approval from South West—Cornwall & Plymouth Research Ethics Committee). All participants (both cases and controls) were aged ≥18 years old and were able to provide informed consent. Any participants who were <18 years old and unable to provide informed consent were excluded from the study.

### Data Collected

All participants (cases, recovered group and healthy group) provided written informed consent before then being asked to complete the Big Five Inventory (BFI) of personality traits, the Generalized Anxiety Disorders-7 (GAD-7) Scale, the Body Vigilance Scale (BVS), the Dizziness Handicap Inventory (DHI), the short form of the Vertigo Symptom Scale (VSS), and the Brief Illness Perception Questionnaire (BIPQ). The BFI consists of 44 questions that assess the five core human personality traits of neuroticism (tendency toward pessimistic worry), extraversion (outgoing nature), openness (to new ideas and experiences), agreeableness (affability and warmth), and conscientiousness (diligence and dutifulness) and provides standardized scores against population norms. Testers respond to the questions with degrees of agreement or disagreement on a five-point Likert scale. For each personality trait, a mean score of higher than 2.5 suggests a tendency toward that trait. The GAD-7 consists of seven questions that measure the severity of state anxiety. The BVS consists of four questions: the first three measure sensitivity and attentiveness to bodily sensations in general; the fourth question measures attentiveness to 15 specific somatic symptoms, including five that are germane to patients with vestibular disorders (dizziness, nausea, faintness, feelings of unreality, and feelings detached from one's self). Testers respond to the questions on an 11 point Likert-like scale ranging from 0 (Not at all like me) to 10 (Extremely like me). A mean score higher than 5.0 suggests a tendency toward strong agreement with the question. The BVS total score is the sum of the answers to questions one to four. The DHI contains 25 questions that measure the severity of handicap due to dizziness-related physical and emotional symptoms and interference with functioning. The short form VSS includes 15 questions, eight that measure severity of vertiginous symptoms and seven that measure severity of associated autonomic/anxiety symptoms. The BIPQ consists of nine questions that assess respondents' understanding, emotional response, and sense of control of their illness, as well as concerns about its causes, consequences, clinical course, and likelihood of treatment response. The DHI, VSS, and BIPQ were administered to the PPPD group only as they were the only ones with active dizziness symptoms required to make sense of the questionnaires.

#### Statistical Analysis (Performed Using SPSS Statistics v26)

Statistical analyses were confined to valid outputs from the questionnaires that tested our hypotheses. As the DHI, VSS, and GAD-7 have not been validated for item-by-item analyses and the BIPQ has no total score, analyses were limited to: BFI—scores for the 5 factors; GAD-7—total score only; BVS—individual item scores; DHI—total score only; VSS—total and two subscale scores; BIPQ—individual items only.

#### BFI, GAD-7, and BVS

Each variable was checked for normality (skewness, kurtosis, Shapiro-Wilk's test), however very few of the variables were normally distributed. Due to this, and the low sample size, non-parametric tests were used. Due to having three independent variables (cases, recovered group, healthy group), an Independent-Samples Kruskal-Wallis test was performed. If a statistically significant result was found, then a *post hoc* pairwise comparison was conducted to determine which of the study groups were statistically different from each other. Statistical significance was determined at *p* < 0.05. Significance values for pairwise comparisons were adjusted using Bonferroni correction for multiple tests and were reported as their adjusted values. Effect sizes, η^2^, were calculated from the Kruskal-Wallis H-value and were interpreted per usual convention as η^2^ < 0.06 (small), 0.06 ≤ η^2^ < 0.14 (medium), and η^2^ ≥ 0.14 (large).

## Results

### Recruitment

#### Cases

The clinical care team at the Department of Otolaryngology, Southend University Hospital NHS Foundation Trust reviewed medical records of current and past patients who were evaluated between January 2019 and January 2020. Eighteen patients who had been given a diagnosis of PPPD in accordance with ICVD criteria (Table 1) during that time period were consecutively asked to take part in the study. All 18 agreed to participate but only 15 completed and returned the questionnaire (see below). These 15 patients were included in the PPPD group with the three non-responders being excluded from the study.

#### Controls

Two sex- and age-matched (±5 years) comparison groups were identified. The first group consisted of patients who had sustained a peripheral vestibular insult but did not develop PPPD (recovered vestibular patient controls). Participants in this group were all consecutively recruited from the dizziness and balance clinic, deemed to have recovered from their vestibular insult and not progressed to PPPD. To be included as a recovered vestibular patient control, patients had to confirm that they had not experienced any vertigo or dizziness within the last year and were currently asymptomatic. As labyrinthine function and symptoms in recovered vestibular patients correlate poorly (15, 16), it was not deemed necessary to subject controls within this group to further vestibular function testing. Confirmation of an asymptomatic state was taken as evidence of full compensation from previous vestibular insult.

The second group consisted of patients from both the general ENT clinic and other non-ENT clinics who had never sustained a vestibular insult (non-dizzy patient controls). This group included any patient who was being followed up for a non-vestibular condition. Patients were asked whether they had ever experienced vertigo and dysequilibrium in the past or whether they had ever been diagnosed with a condition that could cause vertigo and only those who had not were asked to participate. The conditions that these patients were being followed up for included rhinitis (*n* = 2), otitis externa (*n* = 1), tinnitus (*n* = 1), epistaxis (*n* = 1), tympanic membrane perforation (*n* = 1), single-sided deafness (*n* = 1), chronic back pain (*n* = 1), knee osteoarthritis (*n* = 1), deep vein thrombosis (*n* = 1), anal polyps (*n* = 1) and COPD (*n* = 1). The purpose of the non-dizzy comparison group was to control for the state of being ill vs. being healthy. The psychological measures that were investigated are potentially affected by overall health status but vary little with respect to specific types of medical illness.

### Demographics

Fifteen PPPD cases (13 (86.7%) females and two males, with a mean age was 63.7 years) were matched with 12 recovered vestibular controls (10 females (83.3%), mean age 63.8 years) and 12 non-dizzy controls (10 females (83.3%), mean age 62.6 years). The mean duration from diagnosis of PPPD was 5.7 months (range: 0−13 months) and the mean duration of PPPD symptoms was 79.5 months (range: 8−300 months). In the PPPD group vestibular neuronitis (VN) and benign paroxysmal positional vertigo (BPPV) were the two most common triggers (both: *n* = 4, 26.7%), followed by Ménière's disease (*n* = 3, 20%), psychological distress (*n* = 3, 20%) and gentamicin-induced vestibular failure (*n* = 1, 6.7%). Of the three patients who developed PPPD following psychological distress, all three cited bereavement of a spouse (*n* = 2) or parent (*n* = 1) as the trigger of their symptoms. One third of the group were on psychiatric medication. (See Table 2) The vestibular insults in the recovered vestibular group were BPPV (*n* = 6, 50%), VN (*n* = 4, 33.3%), Ménière's disease (treated with intratympanic gentamicin therapy; *n* = 1) and vestibular schwannoma (managed with stereotactic radiotherapy; *n* = 1).

**Table 2:** Demographic data of cases with PPPD.

**Cases**	**Age**	**Sex**	**Duration of PPPD symptoms (months)**	**Precitipating condition**	**Psychiatric medications**
1	78	F	300	BPPV	None
2	34	F	10	Vestibular neuronitis	None
3	67	F	14	Vestibular neuronitis	Fluoxetine
4	69	M	21	Psychological distress	None
5	69	F	8	Psychological distress	Sertraline
6	82	F	14	BPPV	None
7	73	F	9	Gentamicin-induced vestibular failure	None
8	69	F	18	Ménière's disease	None
9	61	F	21	Vestibular neuronitis	None
10	59	F	300	Ménière's disease	Amitriptyline, duloxetine, quetiapine
11	63	F	132	Psychological distress	None
12	49	F	108	Ménière's disease	None
13	47	F	216	BPPV	Fluoxetine
14	70	M	14	BPPV	Sertraline
15	65	F	8	Vestibular neuronitis	None
Mean	**62.6**		**79.5**		

### Big Five Inventory

There was a statistically significant difference between the three study groups in neuroticism (*p* = 0.01; η^2^ = 0.16, large effect), extroversion (*p* = 0.01, η^2^ = 0.20, large effect), and conscientiousness (*p* = 0.03; η^2^ = 0.14, large effect), but not openness (*p* = 0.4). Agreeableness trended toward a significant difference (*p* = 0.09) with a low medium effect; η^2^ = 0.08. Pairwise comparison revealed that these results were largely due to differences between patients with PPPD and non-dizzy controls. Patients with PPPD had higher neuroticism than non-dizzy controls (*p* = 0.02; η^2^ = 0.43, large effect) whilst non-dizzy controls were more extroverted (*p* = 0.008; η^2^ = 0.49, large effect) and more conscientious (*p* = 0.03; η^2^ = 0.41, large effect) than patients with PPPD. A pairwise comparison between patients with PPPD and recovered vestibular controls for neuroticism approached significance (*p* = 0.05). The effect size, η^2^ = 0.38, was large indicating a clinically meaningful difference that a larger sample would have revealed as statistically significant) (See Table 3).

**Table 3:** Big Five Inventory–Patients with PPPD patients and comparison groups.

**Domain**	**Group**	**Mean**	**Std. Error**	**Mean Ranks**	**Kruskal-Wallis test Sig. (*p*)**	**Pairwise Sample**	**Sig. (*p*)**	Adj. sig^*^ (*p*)
Agreeableness	PPPD (15)	3.9	0.13	15.63	0.09	RC^a^-HC^b^	–	–
	RC (12)	4.1	0.15	20.2		RC-PPPD	–	–
	HC (12)	4.4	0.2	27.1		HC-PPPD	–	–
Extraversion	PPPD (15)	2.7	0.2	13.9	0.01	RC-HC	0.15	0.46
	RC (12)	3.4	0.2	20.5		RC-PPPD	0.13	0.4
	HC (12)	3.8	0.1	27.1		HC-PPPD	**0.003**	**0.008**
Conscientiousness	PPPD (15)	3.6	0.2	14.2	0.03	RC-HC	0.5	1.0
	RC (12)	4.1	0.1	22.0		RC-PPPD	0.08	0.23
	HC (12)	4.2	0.1	25.3		HC-PPPD	**0.01**	**0.03**
Openness	PPPD (15)	3.2	0.2	17.3	0.4	RC-HC	–	–
	RC (12)	3.4	0.2	19.9		RC-PPPD	–	–
	HC (12)	3.6	0.2	23.5		HC-PPPD	–	–
Neuroticism	PPPD (15)	3.5	0.2	26.8	0.01	RC-HC	0.8	1.0
	RC (12)	2.6	0.2	16.4		RC-PPPD	**0.02**	**0.05**
	HC (12)	2.5	0.2	15.1		HC-PPPD	**0.008**	**0.02**

* *significance values adjusted by Bonferroni correction for multiple tests*.

a *RC–Recovered Controls (recovered vestibular patients)*.

b *HC–Healthy Controls (non-dizzy patients)*.

*The bold values represent statistically significant results*.

### Generalized Anxiety Disorders-7

With respect to the overall GAD-7 score, there was a statistically significant difference among groups (*p* < 0.02; η^2^ = 0.18, large effect). The mean score for PPPD cases was 10.6; for recovered vestibular controls was 4.6; for non-dizzy controls was 4.1. Pairwise analysis showed that PPPD cases were statistically significantly more anxious than non-dizzy controls (*p* = 0.02; η^2^ = 0.43, large effect) and trended toward a difference from recovered vestibular controls (*p* = 0.07); Again, a large effect size, η^2^ = 0.49, suggested a clinically meaningful difference that missed statistical significance due to the sample size.

### Body Vigilance Scale

There was a statistically significant difference among the study groups in the scores of two subsets of question 4 of the BVS pertaining to how much attention was paid to specific body sensations: feelings of dizziness (*p* < 0.0001; η^2^ = 0.58, large effect) and unreality (*p* = 0.02; η^2^ = 0.15, large effect). In pairwise comparisons, PPPD cases were found to pay more attention to feelings of dizziness than both control group (vs. recovered vestibular controls: *p* = 0.002, η^2^ = 0.53, large effect; vs. non-dizzy controls: *p* < 0.0001, η^2^ = 0.72, large effect) and to feelings of unreality than non-dizzy controls: *p* = 0.02, η^2^ = 0.36, large effect) (See Table 4).

**Table 4:** Body Vigilance Scale (BVS)–Patients with PPPD patients and comparison groups.

	**Group**	**Mean**	**Std. Error**	**Mean Ranks**	**Kruskal-Wallis test Sig. (*p*)**	**Pairwise Sample**	**Sig. (*p*)**	Adj. sig^*^ (*p*)
Q1. “I am the kind of person who pays close attention to internal body sensations.”	PPPD (15)	6.1	0.5	25.9	0.04	RC-HC	0.92	1.0
	RC^a^ (12)	3.5	0.9	16.1		RC-PPPD	0.03	0.08
	HC^b^ (12)	3.6	1.0	16.5		HC-PPPD	0.03	0.1
Q2. “I am very sensitive to **changes** in my internal body sensations.”	PPPD (15)	5.5	0.7	23.7	0.3	RC-HC	–	–
	RC (12)	3.5	0.9	17.0		RC-PPPD	–	–
	HC (12)	4.0	1.2	18.4		HC-PPPD	–	–
Q3. On average, **how much time** do you spend each day scanning your body for sensations?	PPPD (15)	29.3	7.3	25.5	0.03	RC-HC	0.9	1.0
	RC (12)	6.7	3.6	16.8		RC-PPPD	0.03	0.09
	HC (12)	9.2	6.1	16.3		HC-PPPD	0.02	0.07
Q4. Rate how much attention you pay to each of the following sensations using this scale:								
Q4.1. heart palpitations	PPPD (15)	3.3	1.0	21.7	0.6	RC-HC	–	–
	RC (12)	2.9	1.0	20.7		RC-PPPD	–	–
	HC (12)	2.2	0.9	17.3		HC-PPPD	–	–
Q4.2. chest pain/discomfort	PPPD (15)	3.5	1.0	23.4	0.2	RC-HC	–	–
	RC (12)	2.4	1.0	19.6		RC-PPPD	–	–
	HC (12)	1.3	0.7	16.2		HC-PPPD	–	–
Q4.3. numbness	PPPD (15)	3.5	1.0	22.9	0.3	RC-HC	–	–
	RC (12)	2.3	0.7	20.3		RC-PPPD	–	–
	HC (12)	1.4	0.8	16.1		HC-PPPD	–	–
Q4.4. tingling	PPPD (15)	3.5	1.0	23.9	0.2	RC-HC	–	–
	RC (12)	1.7	0.6	17.6		RC-PPPD	–	–
	HC (12)	1.7	0.8	17.5		HC-PPPD	–	–
Q4.5. shortness of breath/smothering	PPPD (15)	4.2	1.1	22.2	0.6	RC-HC	–	–
	RC (12)	2.8	1.0	18.3		RC-PPPD	–	–
	HC (12)	3.0	1.0	19.0		HC-PPPD	–	–
Q4.6. faintness	PPPD (15)	3.6	1.0	23.5	0.2	RC-HC	–	–
	RC (12)	2.0	0.7	19.7		RC-PPPD	–	–
	HC (12)	1.4	0.9	16.0		HC-PPPD	–	–
Q4.7. vision changes	PPPD (15)	4.7	0.8	23.5	0.3	RC-HC	–	–
	RC (12)	2.9	0.8	17.3		RC-PPPD	–	–
	HC (12)	3.2	1.1	18.3		HC-PPPD	–	–
Q4.8. feelings of unreality	PPPD (15)	3.1	0.9	25.1	0.02	RC-HC	0.4	1.0
	RC (12)	0.8	0.4	18.5		RC-PPPD	0.08	0.2
	HC (12)	0.8	0.8	15.1		HC-PPPD	**0.008**	**0.02**
Q4.9. feeling detached from self	PPPD (15)	3.2	0.9	24.6	0.1	RC-HC	–	–
	RC (12)	0.7	0.3	17.3		RC-PPPD	–	–
	HC (12)	1.7	1.0	17.0		HC-PPPD	–	–
Q4.10. dizziness	PPPD (15)	8.2	0.5	30.2	0.0	RC-HC	0.3	0.8
	RC (12)	2.2	0.9	16.0		RC-PPPD	**0.001**	**0.002**
	HC (12)	0.8	0.8	11.2		HC-PPPD	**0.0**	**0.0**
Q4.11. hot flash	PPPD (15)	3.7	1.0	22.9	0.2	RC-HC	–	–
	RC (12)	1.3	0.8	15.8		RC-PPPD	–	–
	HC (12)	3.1	1.0	20.6		HC-PPPD	–	–
Q4.12. sweating/clammy hands	PPPD (15)	2.7	0.9	21.7	0.7	RC-HC	–	–
	RC (12)	1.5	0.8	18.1		RC-PPPD	–	–
	HC (12)	2.3	0.9	19.8		HC-PPPD	–	–
Q4.13. upset stomach	PPPD (15)	4.1	0.9	23.7	0.2	RC-HC	–	–
	RC (12)	1.9	0.8	15.7		RC-PPPD	–	–
	HC (12)	3.2	1.0	19.6		HC-PPPD	–	–
Q4.14. nausea	PPPD (15)	3.5	0.8	24.1	0.1	RC-HC	–	–
	RC (12)	2.8	1.0	20.2		RC-PPPD	–	–
	HC (12)	1.1	0.7	14.6		HC-PPPD	–	–
Q4.13. choking/throat closing	PPPD (15)	4.0	1.0	24.8	0.1	RC-HC	–	–
	RC (12)	1.8	0.9	17.9		RC-PPPD	–	–
	HC (12)	1.3	0.8	16.1		HC-PPPD	–	–

* *significance values adjusted by Bonferroni correction for multiple tests*.

a *RC–Recovered Controls (recovered vestibular patients)*.

b *HC–Healthy Controls (non–dizzy patients)*.

*The bold values represent statistically significant results*.

### Brief Illness Perception Questionnaire

Patients with PPPD agreed quite strongly that PPPD affected their life severely and generally felt that it would last a long time. There was a slightly weaker tendency for patients to indicate feeling like they had no control over their illness and that the treatment they were on for PPPD was unlikely to help their symptoms. There were stronger tendencies for patients with PPPD to consider that their symptoms were severe, concerning, and affecting them emotionally. However, they tended to agree that they understood their illness. When asked to rank the three most likely factors causing their problem, only one third of the patients listed psychological problems, although the wording could have been interpreted as the initial trigger rather than the current mechanism of the symptoms (Table 5).

**Table 5:** Brief Illness Perception Questionnaire (BIPQ)–descriptive statistics for PPPD cases.

	***N***	**Mean**	**Std. Deviation**
Q1. How much does your illness affect your life? (0 = no affect at all, 10 = severely affects my life)	15	7.4	2.6
Q2. How long do you think your illness will continue? (0 = a very short time,; 10 = forever)	14	8.5	2.4
Q3. How much control do you feel you have over your illness? (0 = absolutely no control, 10 = extreme amount of control)	15	4.1	4.0
Q4. How much do you think your treatment can help your illness? (0 = not at all; 10 = extremely helpful)	14	4.2	3.0
Q5. How much do you experience symptoms from your illness? (0 = no symptoms at all; 10 = many severe symptoms)	15	6.3	2.9
Q6. How concerned are you about your illness? (0 = not at all concerned; 10 = extremely concerned)	15	6.8	3.3
Q7. How well do you feel you undertand your illness? (0 = don't understand at all, 10 = understand very clearly)	15	6.1	3.2
Q8. How much does your illness affect you emotionally? (0 = not at all affected emotionally; 10 = extremely affected emotionally)	15	6.9	2.9
Q9. List in rank order the three most important factors that you believe caused your illness	Factor 1	Factor 2	Factor 3
Case 1	Blocked ears	Vertigo	–
Case 2	Heart problem	Low BP	Head injury
Case 3	Labyrinthitis	–	–
Case 4	Anxiety	Poor hearing	Anger
Case 5	Grief	Depression	Anxiety
Case 6	–	–	–
Case 7	Gentamicin	–	–
Case 8	Stress	Tiredness	Migraines
Case 9	Viral infections	Labyrinthitis	Stress
Case 10	Head trauma	Disastrous life	–
Case 11	Mother's death	Father's cancer	Quit smoking
Case 12	Ménière's disease	–	–
Case 13	Labyrinthitis	–	–
Case 14	–	–	–
Case 15	Thunderclap headaches	Delay in BPPV treatment	–

### DHI and VSS

The mean total DHI score was 29.3/100 (range: 11−43, s.d. = 9.8), which is at the upper border of the mild range (0−30) on this questionnaire [Whitney et al. (17)]. The mean total VSS score was 28.7 (range: 10−49; s.d. = 10.8).

### *Post-hoc* Analysis

A *post-hoc* analysis was performed on the data set for BFI, GAD-7, and BVS after removing cases who developed PPPD following psychological trauma (*n* = 3), leaving a more homogeneous subgroup of 12 patients who developed PPPD following peripheral vestibular illnesses, in total (11 females, one male; mean age 62.8 years; mean months from diagnosis, six; mean duration of symptoms, 86 months). All controls (12 recovered vestibular controls and 12 non-dizzy controls) were kept in the data set. The focus of this sub-analysis was therefore on structural vestibular triggers of PPPD likely to present to the ENT surgeon. The statistical treatment of this data set was identical to that of the original data set. The *post-hoc* analysis showed a statistically significant result in one question of the BVS only: PPPD patients were found to pay more attention to feelings of dizziness than both control groups (vs. recovered vestibular controls: *p* = 0.007; vs. non-dizzy controls: *p* < 0.0001). There were no statistically significant differences between patients who developed PPPD after a vestibular illness and either control group with respect to the BFI or GAD-7 questionnaires.

## Discussion

The processes thought to give rise to and then drive PPPD are a combination of those described for its precursors, namely phobic postural vertigo, space-motion discomfort, visual vertigo and chronic subjective dizziness (1). Anxiety and anxiety-related personality traits, in particular neuroticism, have been described as possible predisposing factors, making the affected individual prone to a hypervigilant state of increased introspective self-monitoring that arises from fear of further attacks of vertigo or the consequences of being dizzy during or following the episode of acute vestibular disease (7, 10, 11, 18–25). Yagi et al. (26) have recently developed a PPPD severity questionnaire (the Niigata PPPD Questionnaire) that reflects the diagnostic criteria of PPPD (26). Even more recently, Powell et al. (27) describe PPPD as a complex neurological condition that includes broad perceptual factors and suggest that some individuals' brains are predisposed to generalized cross-modal sensory-overload, giving rise to vulnerability to severe PPPD should a vestibular insult occur (27). What remains to be determined is whether pre-existing psychological risk factors can help in predicting who might be at risk of developing PPPD after an acute vestibular injury, thus allowing for the institution of early treatment (3).

Neuroticism is thought to be one of the key risk factors for the development of PPPD and refers to relatively stable tendencies to respond with negative emotions to threat, frustration or loss (28). Individuals who score highly on the BFI for neuroticism are more prone to anxiety amongst other negative emotions. In a functional MRI (fMRI) study by Indovina et al. (29) it was shown that reduced activation in human analogs of the parieto-insular vestibular cortex (PIVC), hippocampus, anterior insula, inferior frontal gyrus and anterior cingulate cortex, as well as connectivity changes among these regions, may be linked to long-term vestibular symptoms in patients with CSD (29). Also in a fMRI study, Ricelli et al. (30) showed that individual differences in neuroticism were significantly associated with changes in the activity and functional connectivity patterns within visuo-vestibular and anxiety-related systems during simulated vertical self-motion (30). Similarly, Passamonti et al. (31) have shown neuroticism to increase the activity and connectivity of neural networks that mediate attention to visual motion cues during vertical motion. They suggest that this mechanism may mediate visual control of balance in neurotic patients with PPPD (31). In our study, PPPD patients were found to be more neurotic than healthy controls. When compared with recovered controls, the result approached significance only, though the effect size calculation indicated that this negative finding many have been a Type II error given our small sample size. Our study also showed PPPD patients to be more introverted and less conscientious than non-dizzy controls, in keeping with previous research findings by Staab et al. (10) with respect to chronic subjective dizziness (10).

Anxiety is a crucial factor in persisting dizziness (4). The prevalence of anxiety in PPPD has been the focus of one of the treatment modalities, namely cognitive behavioral therapy (CBT) (32). Toshishige et al. (33) have recently demonstrated in a study of 34 patients with PPPD that the presence of comorbid anxiety disorders predicted a considerable improvement of DHI scores from pre-treatment to 6-month following CBT (33). Our data showed a significant difference in anxiety levels between PPPD cases and non-dizzy controls. The comparison with recovered vestibular controls did not reach statistical significance, but again the effect size pointed to a Type II error.

It is interesting that the primary comparisons of all PPPD cases to recovered vestibular controls and the *post-hoc* analysis limited to cases of PPPD following vestibular illnesses found no significantly greater neuroticism or anxiety in those with PPPD. It may be that in a larger cohort, both factors would be significantly higher in PPPD.

Alongside anxiety, a high BVS in the setting of acute vestibular disorders has been shown to predict persistent PPPD-like dizziness far better than measures of structural vestibular deficits (4–7, 32). In a prospective longitudinal study, Heinrichs et al. (5) assessed fear of bodily sensations and cognitions related to anxiety at the time of hospital admission and 3 months later in 43 patients with an episode of VN or BPPV. They showed that the interaction between fear of bodily sensations within the first 2 weeks after admission and the type of vestibular disorder predicted the extent of dizzy complaints 3 months later (5). Our study reflects these findings, with attention to a feeling of dizziness being found to be highly statistically significant in PPPD with respect to the BVS when compared with both control groups. Our *post-hoc* analysis also supports this notion by demonstrating the importance of heightened body vigilance even in a group of patients developing PPPD after vestibular insult.

There is evidence in other areas of medicine that supports the notion that negative illness perception is independently linked to all-cause mortality and can strongly influence recovery from illness which can be slower than in other patients (34–36). Illness perception is seen as an important and potentially modifiable risk factor to target in future disease interventions and intervention has already been shown to reduce illness anxiety, which has relevance in this study (35, 37). One interesting finding in our study is that despite PPPD patients being found to be more neurotic and anxious than healthy controls, only one third of them felt that psychological factors were contributing to their symptoms. This shows similarities to other functional disorders. In a controlled study of 107 patients with functional weakness, Stone et al. (38) showed that these patients tend to reject psychological factors as potentially causal factors (38). They also demonstrated similar findings, though to a lesser degree, in patients with non-epileptic seizures (39). In PPPD, one potential for this finding may be due to the fact that whilst patients experience their dizziness most of the time, they may not necessarily attribute their symptoms to anxiety-related factors or may consider anxiety to be a secondary consequence rather than a contributor to their symptoms. Stigmatization of mental health issues could also play a role, especially in male patients. Targeted patient education on the central role anxiety plays in PPPD could help in addressing this misperception and improving illness perception.

The results of this study support hypotheses derived from investigations of the predecessors of PPPD that anxiety-related factors play important roles in promoting the development of the disorder following conditions that cause vestibular symptoms or disturb balance function, including acute vestibular disorders. However, these results offer a sharper focus, suggesting specifically that heighted body vigilance about dizziness and adverse perceptions of illness may distinguish patients likely to develop PPPD from those more likely to recover from acute illnesses without clinically significant sequelae. The ultimate goal of this line of research is to develop a risk profile that can be used reliably to identify patients susceptible to PPPD so that they may receive early and hopefully preventative interventions. Such a profile is likely to consist of clinical variables present at the time of an acute vestibulopathy (e.g., anxiety-related personality traits, state anxiety) and ones that emerge in the immediate aftermath of acute illness before the onset of chronic morbidity (e.g., adverse illness perceptions).

### Study Limitations

The participant numbers in this exploratory study were small, so no conclusions may be drawn from the results. However, the investigation accomplished its stated objective by gathering pilot data from patients explicitly diagnosed with PPPD to inform the design of more definitive investigations of risk factors and potential early indicators of the disorder.

The study was retrospective and carried with it the inherent problems associated with retrospective studies. Whilst a systematic data collection method was employed, it was collected from patients after they had developed PPPD and at differing times from the onset, thus representing a heterogeneous group.

In our main analysis, PPPD cases were included regardless of their initiating insult, vestibular or otherwise, despite all members of the recovered group having a history of vestibular insult only. This is because the ICVD criteria do not sub-categorize PPPD by type of precipitating event. Our *post-hoc* analysis suggested that this may have had an effect on our results as the comparisons limited to patients who developed PPPD following a vestibular disorder identified a narrower range of differences than the full PPPD cohort compared to recovered controls. Potential differences in risk factors for the development of PPPD following different precipitants merits future study.

Interestingly, our PPPD group was older and consisted of more women than most other reports of PPPD and CSD. This may reflect differences in referrals patterns to various clinical centers around the world and might make our data uncomparable with other studies.

## Conclusion

The data gathered in this pilot study support the design and conduct of fully powered prospective investigations of neuroticism, state anxiety, body vigilance and aberrant illness perceptions as risk factors and contributors to the onset of PPPD that could be formulated into a risk profile to be used for early detection of the disorder in clinical practice.

## Data Availability Statement

The raw data supporting the conclusions of this article will be made available by the authors, without undue reservation.

## Ethics Statement

The studies involving human participants were reviewed and approved by South West – Cornwall & Plymouth Research Ethics Committee. The patients/participants provided their written informed consent to participate in this study.

## Author Contributions

AT was the principal investigator and responsible for the study design, conducting the study, data collection, and preparation of the manuscript. PH was responsible for liaison with study participants, liaising with research and development and ethics departments, conducting the study, supervision of study flow, data processing and analysis and manuscript editing, and proofreading. JS, JPS, and JG were responsible for study design, supervision of the study and manuscript editing, and proofreading. All authors contributed to the article and approved the submitted version.

## Conflict of Interest

The authors declare that the research was conducted in the absence of any commercial or financial relationships that could be construed as a potential conflict of interest.
